# Supplementary material for: Peroxisome Proliferator-Activated Receptor Gamma (PPARγ) Suppresses Inflammation and Bacterial Clearance during Influenza-Bacterial Super-Infection
Source: Viruses. 2019 Jun 1;11(6):505. doi: 10.3390/v11060505 (PMC6630660; doi:10.3390/v11060505)
Supplement: Supplementary file 1 [file viruses-11-00505-s001.pdf]

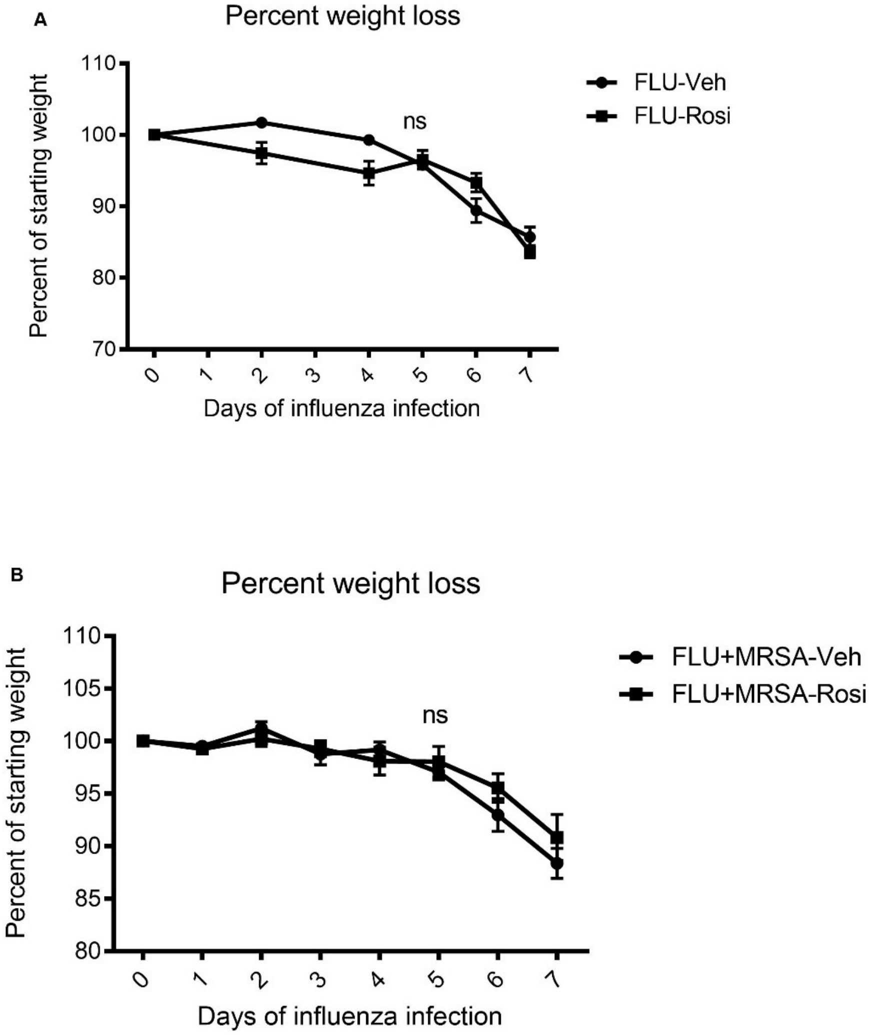

Figure S1. Rosiglitazone treatment has no impact on weight loss during influenza and influenza-MRSA super-infection. WT male, 6–8 weeks old mice were infected with 100 pfu of influenza or influenza and MRSA super-infected as described in methods (*N* = 7–8 per group). Mice were treated with rosiglitazone or vehicle (DMSO) from day 0-6 days post-infection and harvested on day 7 post-infection. (**A**) Percentage weight loss was measured during influenza infection, (**B**) influenza-MRSA super-infection. Data are represented as mean ± SEM, two tailed Student's *t* test, ns-not significant.
